# Novel non-invasive intrarenal pressure monitoring devices in flexible ureteroscopy: an in-vitro comparative study

**DOI:** 10.1007/s00345-025-05761-8

**Published:** 2025-06-30

**Authors:** Steffi Kar Kei Yuen, Zixiao Chen, Skyler Yuen, Alex Qinyang Liu, Chi-Ho Leung, Ivan Ching Ho Ko, Chi Kwok Chan, Bhaskar Somani, Thomas Herrmann, Helene Jung, Theodoros Tokas, Stefanie Croghan, Olivier Traxer, Peter Ka Fung Chiu, Ben Chew, Jeremy Yuen Chun Teoh, Vineet Gauhar, Zheng Li, Chi Fai Ng

**Affiliations:** 1 S.H. Ho Urology Centre, Department of Surgery, The Chinese University of Hong Kong, Hong Kong, China; 2 European Association of Urology Section of Endourology (ESEUT), Arnhem, The Netherlands; 3 Multi-scale Medical Robotics Center, Chow Yuk Ho Technology Centre for Innovative Medicine, The Chinese University of Hong Kong, Hong Kong, China; 4 Cheltenham Ladies College, Cheltenham, UK; 5 Department of Urology, NHS Trust, University Hospital Southampton, Southampton, UK; 6 Department of Urology, Kantonspital Frauenfeld, Spital Thurgau AG, Frauenfeld, Switzerland; 7 Urological Research Center, Department of Urology, Vejle Hospital (a part of Lillebaelt Hospital), University Hospital of Southern Denmark, Vejle, Denmark; 8 Department of Regional Health Research, University of Southern Denmark, Odense, Denmark; 9 Department of Urology, Medical School, University General Hospital of Heraklion, University of Crete, Heraklion, Greece; 10 Training and Research in Urological Surgery and Technology (T.R.U.S.T.)-Group, Hall in Tirol, Austria; 11 Department of Surgery, Royal College of Surgeons in Ireland, Dublin, Ireland; 12 Department of Urology, Freeman Hospital, Newcastle, UK; 13 Department of Urology, Sorbonne University, GRC n°20 Lithiase Renale, AP-HP, Hôpital Tenon, 75020 Paris, France; 14 Department of Urology, University of British Columbia, Vancouver, Canada; 15 Li Ka Shing Institute of Health Sciences, The Chinese University of Hong Kong, Hong Kong, China; 16 Department of Urology, Medical University of Vienna, Vienna, Austria; 17 Department of Urology, Ng Teng Fong Hospital, Singapore, Singapore; 18 Department of Surgery, Multi-scale Medical Robotics Center, Chow Yuk Ho Technology Centre for Innovative Medicine, The Chinese University of Hong Kong, Hong Kong, China

**Keywords:** Flexible and navigable Suction ureteral access sheath, Intrarenal pressure, Optical fibre pressure sensor, Renal pelvic pressure, Ureteroscopy

## Abstract

**Purpose:**

Intrarenal pressure monitoring is emerging as an important tool in flexible ureteroscopy. Novel non-invasive intrarenal pressure monitoring devices are introduced. We evaluated the pressure readings simultaneously across four novel non-invasive intrarenal pressure monitoring devices against the urodynamic system.

**Materials and methods:**

Two IRP monitoring single-use flexible ureteroscopy: LithoVue Elite^™^ (Boston Scientific Corporation, Marlborough, MA, USA); ZebraScope^™^ (Happiness Works Medical Instruments Co., Ltd, Anhui, China); two intelligent intrarenal system integrated with irrigation and suction via flexible and navigable suction ureteral access sheath: i-MIMERsys^™^ (ZSR Biomedical Technology Co., Ltd, Dongguan, China); Tidor^™^ (YiGao Medical Technology Co., Ltd, Zhejiang, China) were tested against MMS/Laborie Nexam Pro Urodynamic System (Laborie Medical Technologies Corporation, Portsmouth, USA).

**Results:**

The degree of agreement between measurements from urodynamic system and the experimented device was demonstrated by Bland–Altman plots and intraclass correlation coefficients (ICCs). Compared to the urodynamic system, the average difference ± standard deviation of LithoVue Elite™ was 0.26 ± 1.75mmHg; ZebraScope™ 3.22 ± 1.86mHg, i-MIMERsys™ 0.96 ± 2.26mmhg; Tidor™ 1.09 ± 2.57mmHg. Pressure measured by LithoVue Elite™, ZebraScope™, i-MIMERsys™ and Tidor™ to urodynamic system showed excellent ICCs of 0.975 (*p* < 0.001), 0.915(*p* = 0.016), 0.955(*p* < 0.001) and 0.937(*p* < 0.001) respectively.

**Conclusion:**

Pressure recordings from four novel non-invasive intrarenal monitoring devices, namely flexible ureteroscopes—LithoVue Elite^™^, ZebraScope^™^; and intelligent IRP monitoring systems via flexible and navigable suction ureteral access sheaths — i-MIMERsys^™^, Tidor^™^. were comparable to that of urodynamic system. This in-vitro study verifies that such non-invasive devices provide reliable data for monitoring intrarenal pressure changes.

## Introduction

Intrarenal pressure (IRP) monitoring is emerging as an essential tool in flexible ureteroscopy (FURS).

Catheter pressure transducers are well established in urology applications like urodynamic studies. Guidewires and catheter pressure transducers operate on the principle of electro-mechanical pressure transducers, often integrating multiple functionalities. According to the working principle, pressure sensors can be classified into piezoresistive, capacitive, optical fiber, resonant, and piezoelectric types [1].

The latest review by the Global Research on Intrarenal Pressure Collaborative Group proposed the classification of IRP monitoring devices into flexible ureteroscopes, flexible and navigable suction ureteral access sheaths (FANS) with intelligent irrigation and suction systems and stents [2] and pressure sensing wires.

Single-use digital pressure-sensing flexible ureteroscope employs optical fibre pressure sensors (OFPS), specifically a Fabry–Perot interferometer. Such miniaturised pressure sensors are inbuilt at the tip of the single-use digital flexible ureteroscopes, and the real-time IRP is displayed alongside the endoscopic view. Bhojani et al. demonstrated clinical utility of a single-use digital flexible ureteroscope with integrated pressure sensors using the Lithovue Elite^™^ (Boston Scientific Corporation, Marlborough, USA) [3].

Recent advancements have enabled real-time IRP monitoring and regulation in adults and children undergoing FURS, utilizing FANS with intelligent IRP system integrated with irrigation and suction [4, 5]. The IRP monitoring FANS resembles the structure of a conventional FANS. A pressure-measuring port is located at the distal tip, which is the opening to a tiny 0.5Fr channel embedded within the wall of the sheath. The IRP monitoring FANS is connected to a piezoresistive silicon pressure sensor that converts the transduced pressure signal into a voltage output at the external end [5]. When placed within the pelvicalyceal system, the IRP is transduced via the pressure-measuring port from the tip of FANS via the tiny embedded channel to the sensor connected externally to the processor system. The real-time IRP is displayed on a monitor, allowing instant observation and regulation by the surgeon.

Currently, there is no urology dedicated pressure sensor guidewire. Doizi et al. utilized a wire sensor designed initially for cardiac pressure monitoring in human studies [6].

IRP monitoring flexible ureteroscopes are able to detect pressure ranging from − 30mmHg to 300mmHg, whereas IRP monitoring FANS integrated with intelligent irrigation and suction platforms are able to detect pressure ranging from − 300mmHg to 300mmHg. Their sampling rates vary from once per second to once per 2.5 millisecond.

With these latest advancements, real-time non-invasive IRP monitoring is now possible during endourological procedures. Although a plethora of novel technologies exists, there is limited comparative performance across different systems [2, 7, 8]. Therefore, it is imperative to evaluate the options for real-world application and understand the variations among novel IRP monitoring devices. We aim to evaluate the variations of pressure readings across novel non-invasive intrarenal pressure monitoring devices when placed under the same conditions and compare it against the time tested urodynamic system.

## Materials and methods

**Fig. 1:**
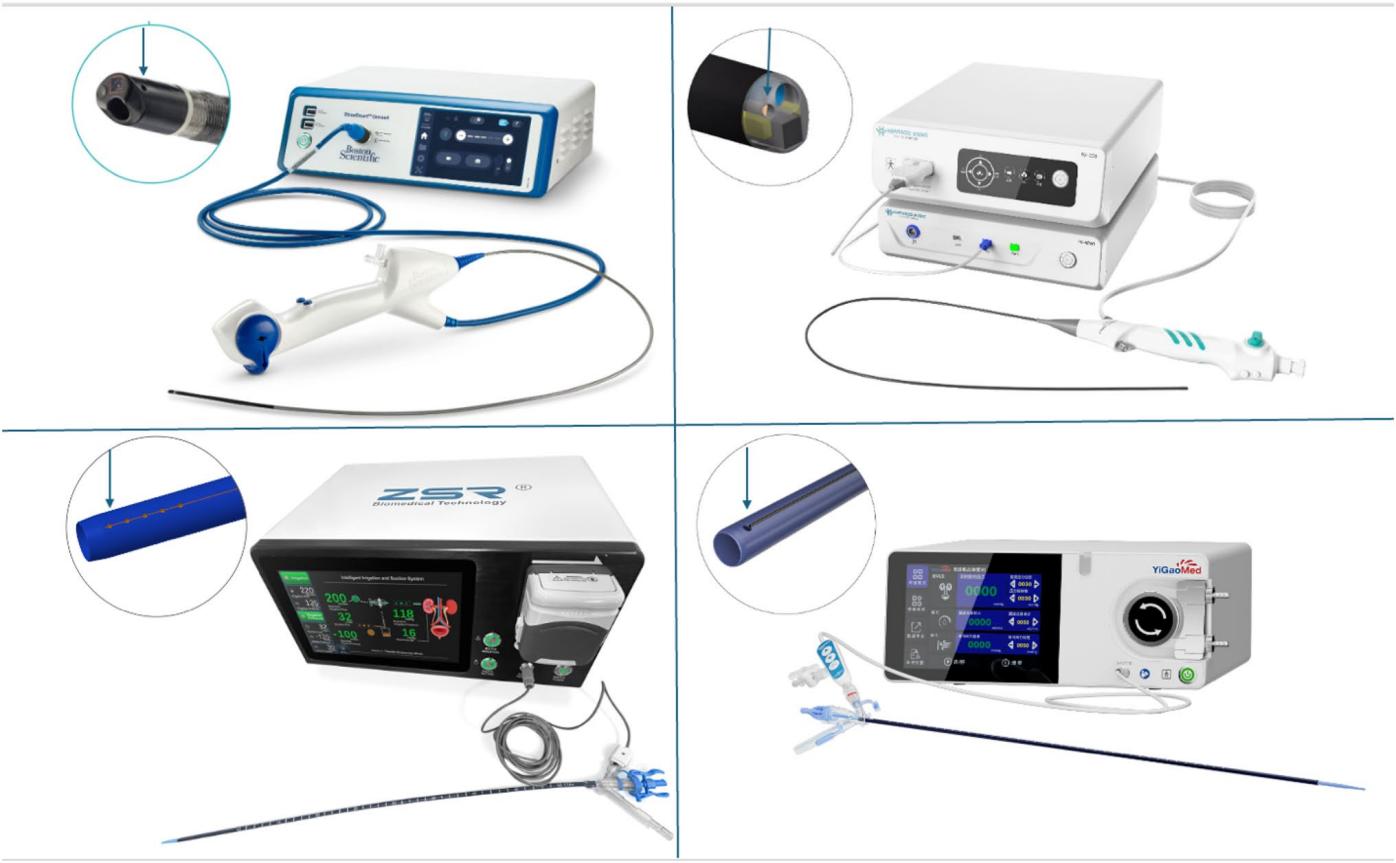
IRP monitoring devices connected to their respective processors with arrows pointing at the location of pressure sensing: LithoVue Elite™ (top left), ZebraScope™ (top right), i-MIMERsys™ (bottom left), Tidor™ (bottom right)

Our study aims to evaluate simultaneously the pressure readings across four commercially available IRP monitoring devices against the conventional urodynamic system. The IRP monitoring devices (Fig. 1) tested include:

1. LithoVue Elite^™^ (LVE)(Boston Scientific Corporation, Marlborough, USA).
2. ZebraScope^™^ (Happiness Works Medical Instruments Co., Ltd, Anhui, China).
3. i-MIMERsys^™^ (ZSR Biomedical Technology Co., Ltd, Dongguan, China).
4. Tidor^™^ (YiGao Medical Technology Co., Ltd, Zhejiang, China).

### Experimental setup (Fig. 2)

**Fig. 2:**
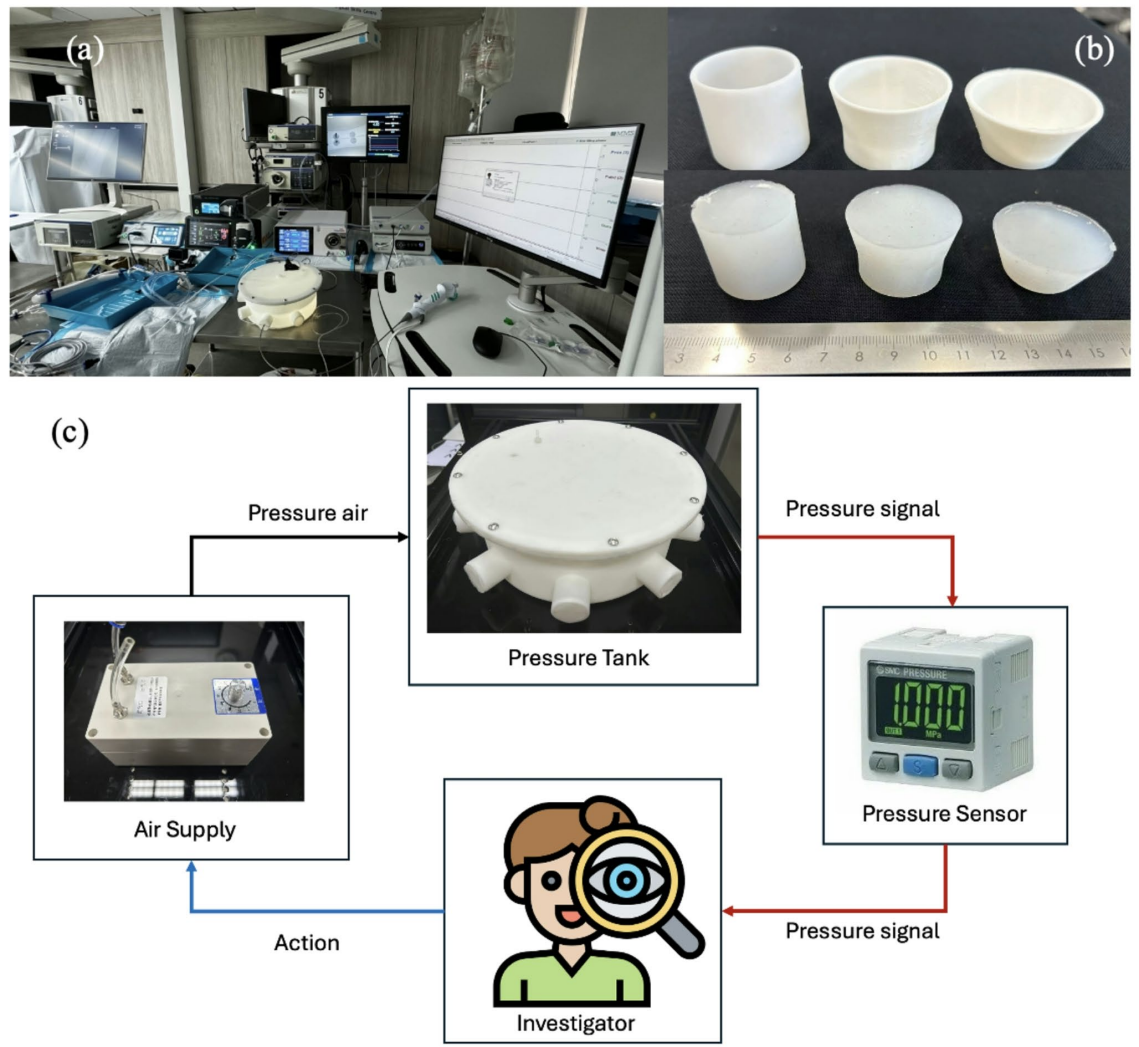
**a** Experimental setup of devices from left to right: LithoVue Elite™, i-MIMERsys™, Tidor™, ZebraScope™, MMS/Laborie Nexam Pro UDS; **b** top: mold for silicon cork used in pressure tank; bottom: silicon cork(ECOFLEX 00–10); **c** Diagram showing experimental workflow: The system operates with a pressure-adjustable pneumatic pump which is manually controlled. This pump outputs pressure to the tank, monitored continuously by a sensor (SMC-ISE20) that provides real-time readings displayed on a digital screen, allowing feedback regulation via a Proportional Integral Derivative (PID) controller

The measurements of the four systems were compared with MMS/Laborie Nexam Pro Urodynamic System (UDS) (Laborie Medical Technologies Corporation, Portsmouth, USA), known for its sensitivity in detecting fluid pressure changes in urodynamic study. The IRP sensing function characteristics of each device were tabulated (Table 1).

**Table 1:** Characteristics of pressure measurement function of the four experimented devices and the urodynamic system

	Device	Location of pressure sensor	Range of pressure measured	Sampling rate	Alarm and intelligence function
MMS/Laborie Nexam Pro Urodynamic System (Laborie Medical Technologies Corporation, Portsmouth, ND, USA)	Urethral catheter connected to external sensor	Connected to the end of ureteric catheter	−50 to 150 cmH2O (i.e. −36.5 to 109.5 mmHg)*	Once per 100ms	None
LithoVue Elite^™^ (LVE) (Boston Scientific Corporation, Marlborough, MA, USA)	9.5Fr single-use digital flexible ureteroscope	Inbuilt in scope tip	−30mmHg to 300mmHg	Once per 250ms	Alarm but no intelligence regulation
ZebraScope™ (Happiness Works Medical Instruments Co., Ltd, Anhui, China)	8.6Fr single-use digital flexible ureteroscope	Inbuilt in scope tip	−30mmHg to 300mmHg	Once per second	Alarm but no intelligence regulation
i-MIMERsys™ (ZSR Biomedical Technology Co.,Ltd, Dongguan, China)	Intelligent IRP system integrated with irrigation, suction via FANS	Connected to the side channel at the end of FANS	−300mmHg to 300mmHg	Once per 2.5ms	Both alarm and intelligence regulation function present
Tidor™ (YiGao Medical Technology Co., Ltd, Zhejiang, China)	Intelligent IRP system integrated with irrigation, suction via FANS	Connected to the side channel at the end of FANS	−30mmHg to 300mmHg	Once per 200ms	Both alarm and intelligence regulation function present

*IRP* intrarenal pressure, *FANS* flexible and navigable suction ureteral access sheath

*(1 cm H2O = 0.73 mm Hg)

A custom-designed 3D-printed circular pressure tank (R4600 Resin) simulates the pelvicalyceal system. Its side ports are sealed with silicon corks, allowing simultaneous placement of pressure-sensing devices while maintaining a pressure and fluid integrity. Fluid enters via a separate opening, and a pressure regulator connected to a pneumatic pump forms the central component of a closed-loop control system. The investigator can adjust the pump to achieve the desired pressure levels, ensuring precise control over the internal pressure during experiments.

A fluid-filled 5-Fr catheter for the urodynamic system, along with the flexible ureteroscopes of (LVE and ZebraScope^™^) and IRP-sensing FANS (i-MIMERsys^™^ and Tidor^™^), were inserted into the tank and held in place 10 cm from its tip at the same height and level by the silicon corks. All devices were calibrated to zero according to the manufacturer’s guidelines.

Subjecting all devices under the same pressure conditions within the pressure tank, positive pressure was exerted by the investigator with the voltage regulator, allowing time in between for the pressure to fall back to its baseline before next pressure exertion.

### Statistical analysis

Bland–Altman plots and the two-way mixed-effects, single measure, absolute agreement intraclass correlation coefficients (ICCs) were used to assess agreement between UDS measurements and those from the experimented device [9–11]. A two-sided p-value < 0.05 was considered statistically significant. All analyses were conducted using R version 4.3.1 (R Foundation for Statistical Computing).

## Results

**Fig. 3:**
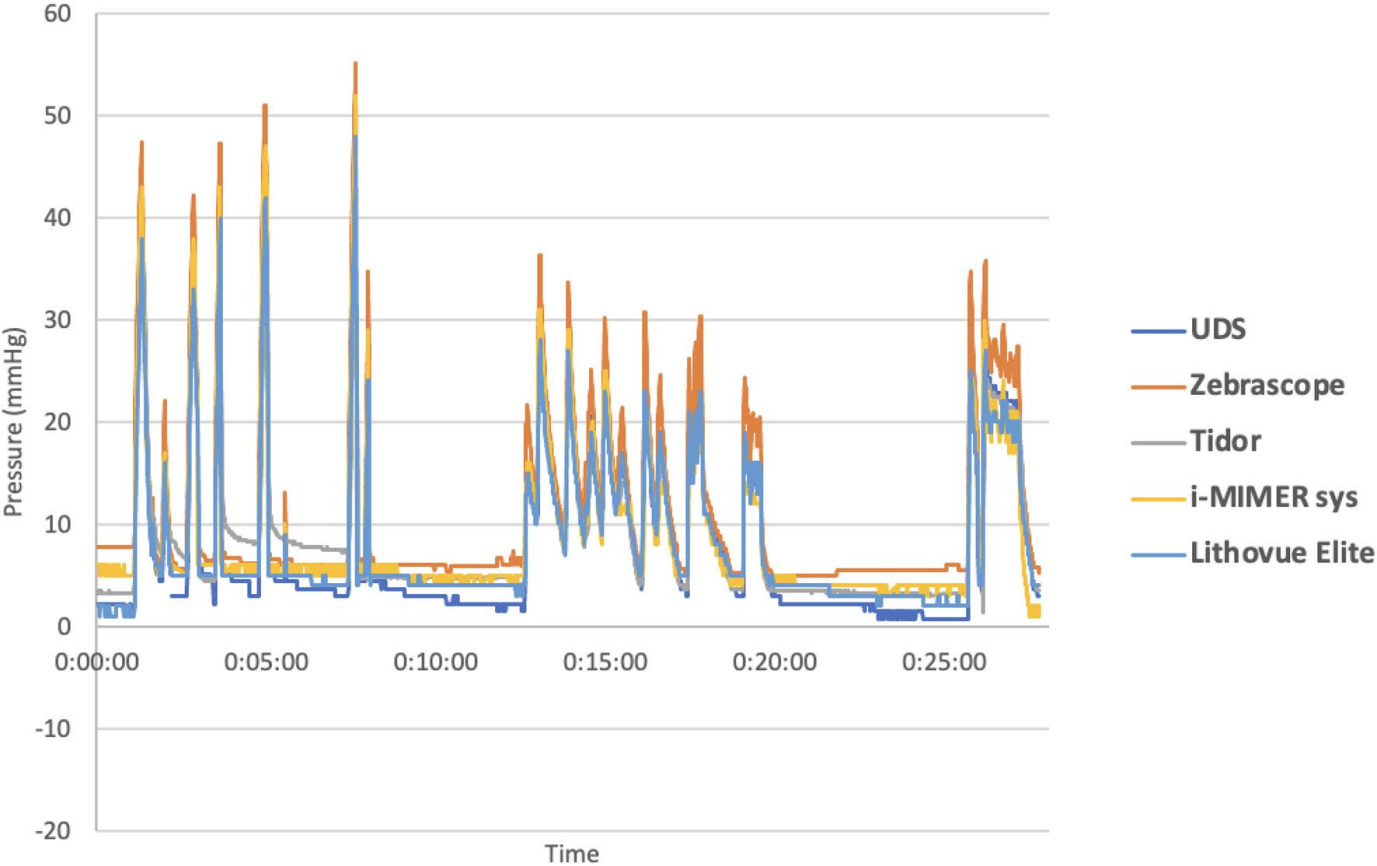
The pressure measurements of the four experimented IRP monitoring devices and UDS against time

Continuous real-time pressure measurements with 19 episodes of pressure generation were recorded, i.e. 16,690 measurements by UDS system, 1669 by LVE, ZebraScope™ and i-MIMERsys™, and 1625 by Tidor™ simultaneously. The pressure curves derived from the respective electronic recordings were charted in Fig. 3.

**Fig. 4:**
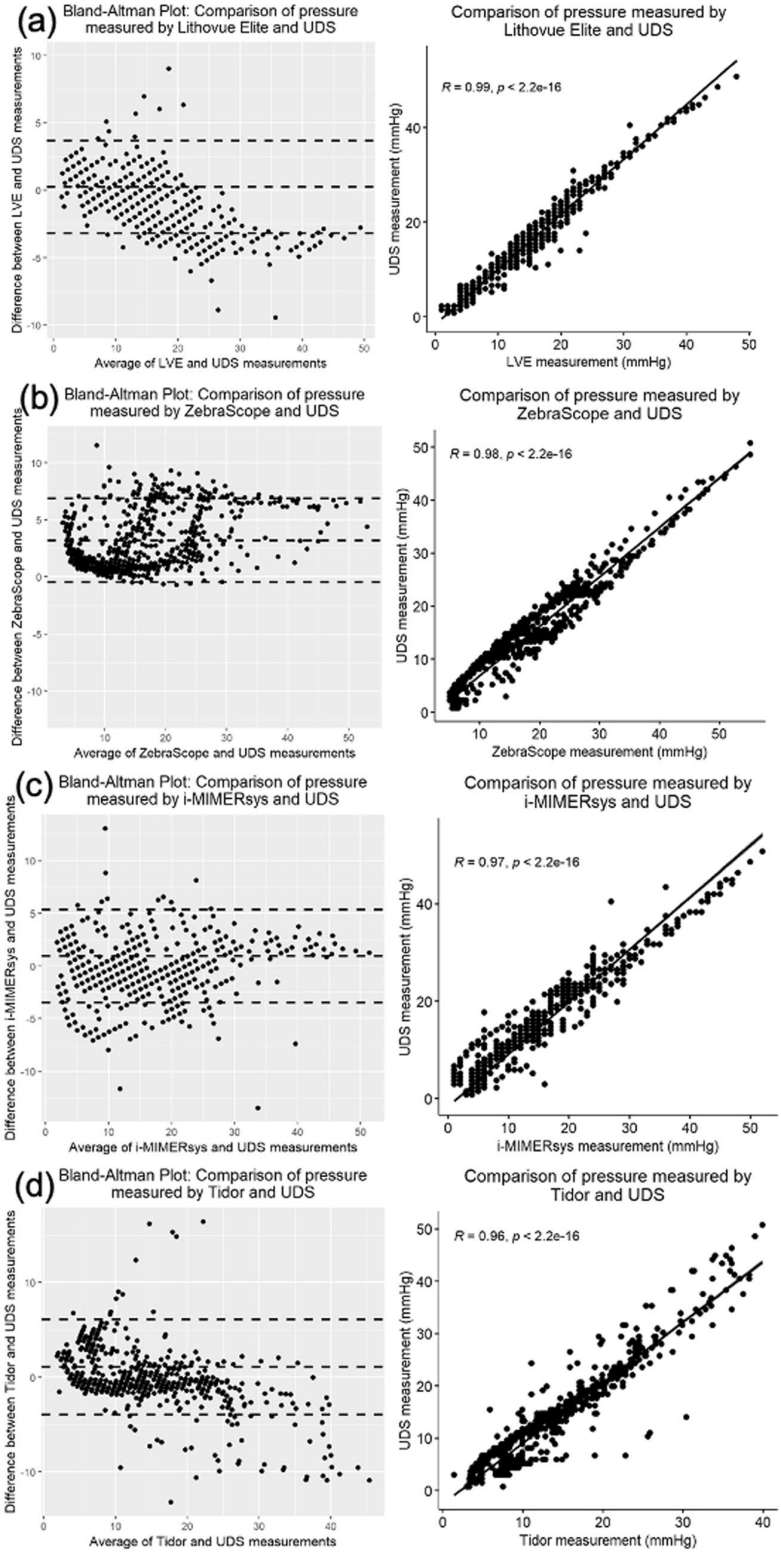
Bland–Altman (BA) plots of the four IRP devices with reference to UDS measurement. **a** LithoVue Elite™, **b** ZebraScope™, **c** i-MIMERsys™, **d** Tidor™

Bland-Altman(BA) plots (Fig. 4) compared pressure measurements using LVE, ZebraScope™, i-MIMERsys™, Tidor™ and UDS, respectively. Compared to the UDS measurement, the average difference ± standard deviation(SD) of LVE was 0.26mmHg ± 1.75mmHg; ZebraScope™ 3.22mmHg ± 1.86mmHg, i-MIMERsys™ 0.96mmHg ± 2.26mmhg; Tidor™ 1.09mmHg ± 2.57mmHg.

ICCs for IRP measured using LVE, Zebrascope™, i-MIMERsys™ and Tidor™ compared to UDS are 0.975(*p* < 0.001), 0.915(*p* = 0.016), 0.955(*p* < 0.001) and 0.937(*p* < 0.001) respectively. (Table 2)

**Table 2:** Agreement between the four IRP devices with reference to UDS measurement

	Average difference (SD)	ICC (95% CI)
LithoVue Elite™	0.26 (1.75)	0.975 (0.972–0.978)
ZebraScope™	3.22 (1.86)	0.915 (0.073–0.977)
i-MIMERsys™	0.96 (2.26)	0.955 (0.928–0.969)
Tidor™	1.09 (2.57)	0.937 (0.902–0.957)

*SD* standard deviation, *ICC* intraclass correlation coefficient, *CI* confidence interval

## Discussion

In-vitro and in-vivo IRP monitoring in endourology were reviewed by Croghan et al. [12]. All studies analysed single IRP monitoring devices under variable setup, lacking standardized outcome reporting. These studies primarily utilized minimally invasive measurements by pressure sensors on cardiology guidewires, nephrostomy tubes, or ureteric catheters connected to urodynamic systems. Each reported different baseline pressures, influenced by irrigation techniques and lacked comparators to assess efficacy. While it paved the way for non-invasive IRP recording, there is limited data on the accuracy of these measurements in reflecting true IRP values.

Previous limitations included pressure sensors at the distal end of sheaths or guidewires that were unable to reflect the actual intrarenal values promptly. Additionally, they could not be placed into the operated calyx in close proximity for manometry, raising concerns about pressure variations due to sensor positioning.

Given the emergence of IRP monitoring devices, we found it compelling to conduct an experiment that standardized simultaneous performance recording of such devices in a controlled setup. No prior studies have explored the comparability of these pressure measurement devices.

Although our experimental design may not perfectly mimic actual human conditions, it allows for homogenous pressure monitoring and direct in-vitro comparisons. Device tips were placed 10 cm towards the center of the tank to eliminate differential pressure gradient. The study results are difficult to emulate simultaneously in cadaveric or perfused animal models in a single setting as these devices cannot be placed synchronously in one kidney.

Further, we selected a well-established, time-tested tool in urodynamic assessment—the MMS/Laborie Nexam Pro Urodynamic System (Laborie Medical Technologies Corporation, Portsmouth, ND, USA), as our reference measurement [13]. Our study demonstrated a strong intraclass correlation (ICC = 0.915–0.975) across all devices with UDS, with average difference of 0.26-3.22mmHg.

Our study does have its limitations. First, being in-vitro, it does not account for real-world clinical factors like ureteral access sheath size, patient positioning, all of which influence the IRP [12, 14–16]. Second, it cannot assess negative pressure which may be present during suction, since UDS cannot detect negative values. Simultaneous measurements of all devices may not be feasible in vivo, future research in an animal model can be explored so as to provide data that may further reflect clinical reality. Future research is required to validate such novel non-invasive IRP monitoring devices in clinical applications.

## Conclusion

Intrarenal pressure (IRP) monitoring is emerging as an essential tool in flexible ureteroscopy. We studied simultaneous pressure measurements from urodynamic system against four novel non-invasive IRP monitoring devices, namely flexible ureteroscopes—LithoVue Elite^™^, ZebraScope^™^; and intelligent IRP monitoring systems via flexible and navigable suction ureteral access sheaths—i-MIMERsys^™^, Tidor^™^. This in-vitro study verifies that such novel non-invasive devices provide reliable data for monitoring intrarenal pressure changes.

## Data Availability

The data that support the findings of this study are available from the corresponding author upon reasonable request.
